# Supplementary figures and images for: Multi-omics approaches for comprehensive analysis and understanding of the immune response in the miniature pig breed
Source: PLoS One. 2022 May 19;17(5):e0263035. doi: 10.1371/journal.pone.0263035 (PMC9119490; doi:10.1371/journal.pone.0263035)

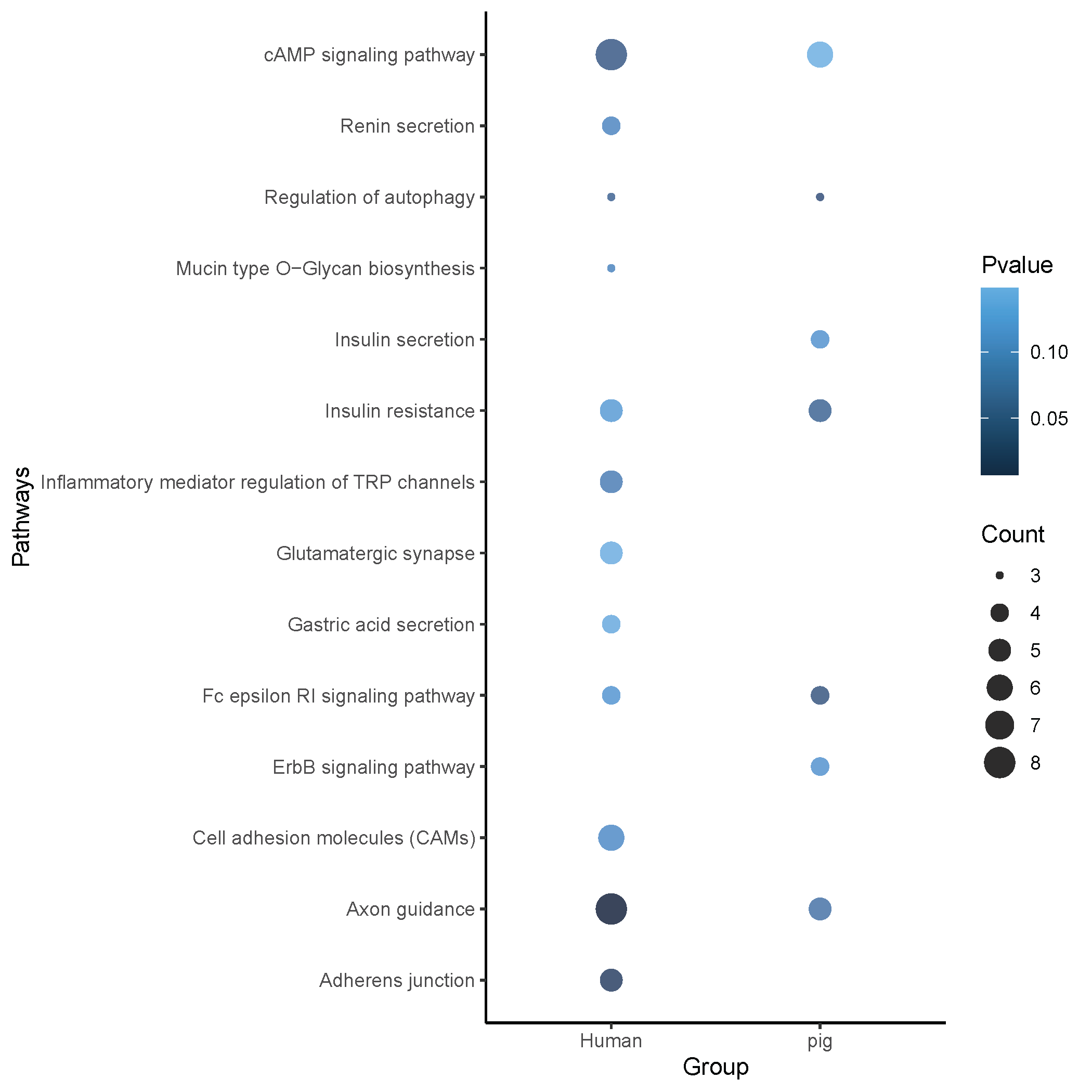

Supplement: S1 Fig — (TIF) [file pone.0263035.s005.tif]

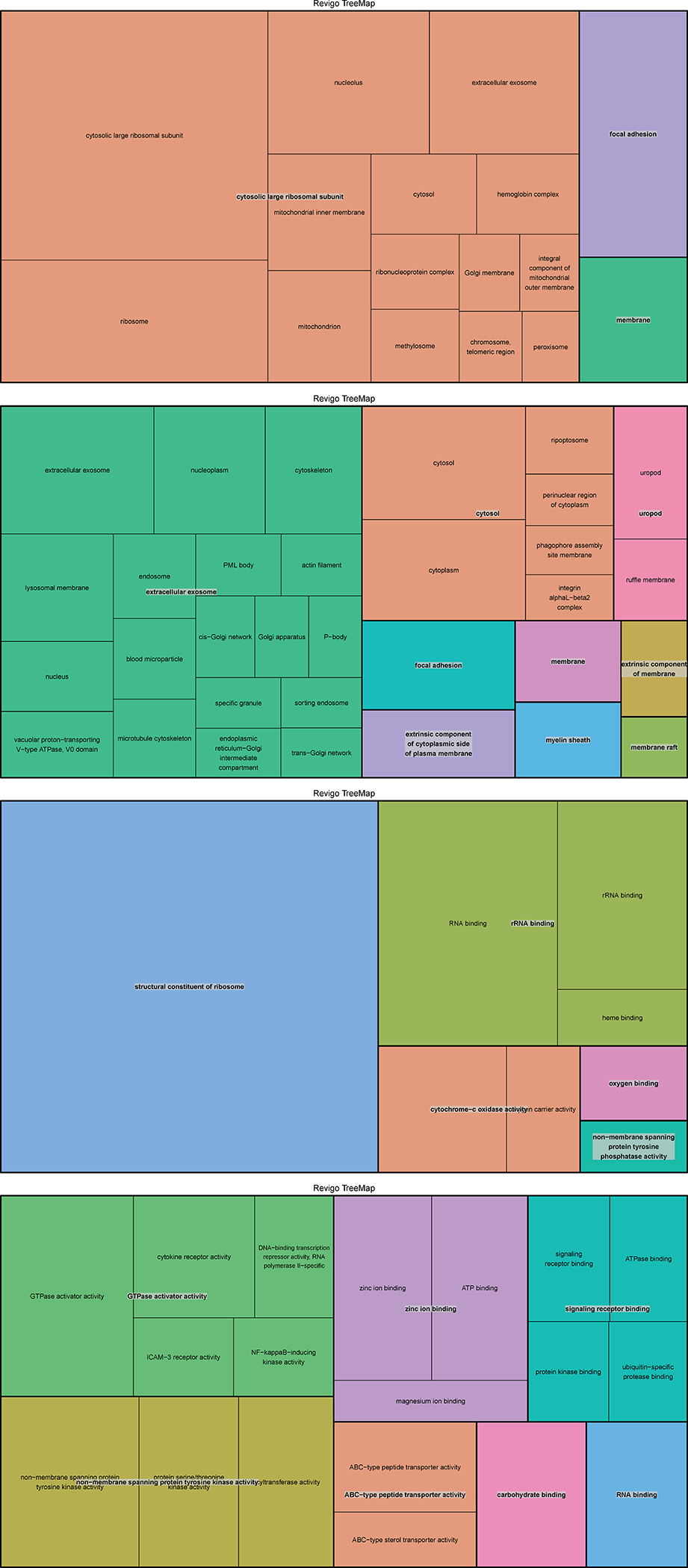

Supplement: S2 Fig — (TIF) [file pone.0263035.s006.tif]
