# Supplementary material for: Multi-omics approaches for comprehensive analysis and understanding of the immune response in the miniature pig breed
Source: PLoS One. 2022 May 19;17(5):e0263035. doi: 10.1371/journal.pone.0263035 (PMC9119490; doi:10.1371/journal.pone.0263035)
Supplement: Attachment — Submitted filename: Comment.docx [file pone.0263035.s007.docx]

Article entitled, “ Multi-omics approaches for comprehensive analysis and understanding of immune response in the immature pig breed” is an unique study and is helpful for future approach for biomedical research. Therefore, I recommend this MS for publication after incorporating very minor corrections:

Line 44: Due to different size …..(the sentence needs correction). In my opinion this should be written as “ Due to different size and varied immune response, the test result is not mimicked in rodents which is otherwise possible in pig models. This has popularized pig for feasible use in biomedical research (6,7).

Line 49: Please delete the word dramatic

Line 50: “Furthermore, the immune system of minipig………preclinical trials.” Please make it two sentences.

Line 56: Why unnecessarily after genetic engineering “Hominidae” has been mentioned in bracket. Authors please justify.
