# Supplementary material for: Multi-omics approaches for comprehensive analysis and understanding of the immune response in the miniature pig breed
Source: PLoS One. 2022 May 19;17(5):e0263035. doi: 10.1371/journal.pone.0263035 (PMC9119490; doi:10.1371/journal.pone.0263035)
Supplement: Attachment — Submitted filename: Response to Reviewers.docx [file pone.0263035.s008.docx]

**Revision: ‘** **Multi-omics approaches for comprehensive analysis and understanding of the Immune response in the Miniature Pig breed’**

**#Editor Comments**

We are very grateful to Editor critical examination and pointing out issues. We have addressed all the concern queries as suggested in the entire manuscript for the same.

**Comment 1:** Please ensure that your manuscript meets PLOS ONE's style requirements, including those for file naming.

**Response:** We are thankful to the editor for highlighting the formatting issue. We have made changes in the manuscript as per PlosOne standard.

**Comment 2:** To comply with PLOS ONE submissions requirements, in your Methods section, please provide additional information on the animal research and ensure you have included details on (1) methods of sacrifice, (2) methods of anesthesia and/or analgesia, and (3) efforts to alleviate suffering.

**Response:** Method section has been updated with the added information and provided the supporting reference used to perform the experiment.

**Comment 3:** We note that you have provided funding information that is not currently declared in your Funding Statement. However, funding information should not appear in the Acknowledgments section or other areas of your manuscript. We will only publish funding information present in the Funding Statement section of the online submission form. Please remove any funding-related text from the manuscript and let us know how you would like to update your Funding Statement.

**Response:** We have removed the funding statement from the main manuscript and provided the same with the cover letter.

**Comment 4:** Your ethics statement should only appear in the Methods section of your manuscript. If your ethics statement is written in any section besides the Methods, please move it to the Methods section and delete it from any other section. Please ensure that your ethics statement is included in your manuscript, as the ethics statement entered into the online submission form will not be published alongside your manuscript.

**Response:** Done as suggested.

**Comment 5:** Additional Editor Comments:
As suggested by both the reviewers the MS in its current from is full of grammatical and syntactical errors. Authors are instructed to edit the MS for English language with the help of either a native English speaker or a reputed English editing service provider (in both cases certificate/proof has to be attached while uploading the revised MS). In addition, provide figures of acceptable quality as suggested by one of the reviewers.

**Response:** We have critically evaluated and used English editing service to improve grammatical errors in the manuscript.

**Reviewer #1:**

**Comment 1:** General comments # 1: There are issues with the usage of English

**Response:** We are thankful to the reviewer for highlighting the concern issue. We have taken this as critical issue and consulted with English expert to fix all the issue related with manuscript writing.

**Comment 2:** General comments # 2: The text does not flow logically in the introduction, results and discussion, thereby greatly hampering the readability.

**Response:** In the revised manuscript we have taken all precaution and re-write the mention section for easy to understand and maintaining the continuity of work flow.

**Comment 3:** The authors have not been careful and critical while drafting the manuscript. This is indicated by the spelling mistakes, undefined abbreviations and grammatical errors etc.

**Response:** Done as suggested.

**Comment 4:** General comments # 4: All the figures should be replaced with good quality images

**Response:** All the figure quality has been updated and replaced.

**Reviewer #2:**

Article entitled, “ Multi-omics approaches for comprehensive analysis and understanding of immune response in the immature pig breed” is an unique study and is helpful for future approach for biomedical research. Therefore, I recommend this MS for publication after incorporating very minor corrections:

We are grateful to the reviewer for critically examine the manuscript and providing the positive feedback for the same. We have incorporated all the changes as suggested by the reviewer in the revised submission.

**Comment 1:** Line 44: Due to different size …..(the sentence needs correction). In my opinion this should be written as “ Due to different size and varied immune response, the test result is not mimicked in rodents which is otherwise possible in pig models. This has popularized pig for feasible use in biomedical research (6,7).

**Response:** Done as suggested.

**Comment 2:** Line 49: Please delete the word dramatic

**Response:** Done as suggested.

**Comment 3:** Line 50: “Furthermore, the immune system of minipig………preclinical trials.” Please make it two sentences.

**Response:** We re-wrote the mention section for clear and understanding.

**Comment 4**: Line 56: Why unnecessarily after genetic engineering “Hominidae” has been mentioned in bracket. Authors please justify.

**Response:** We agreed with the reviewer comment and remove the same from the manuscript.
